# Capillary Sodium Dodecyl Sulfate Agarose Gel Electrophoresis of Proteins

**DOI:** 10.3390/gels8020067

**Published:** 2022-01-19

**Authors:** Daniel Sarkozy, Andras Guttman

**Affiliations:** 1 Horváth Csaba Memorial Laboratory of Bioseparation Sciences, Research Center for Molecular Medicine, Faculty of Medicine, Doctoral School of Molecular Medicine, University of Debrecen, 98 Nagyerdei Krt, H-4032 Debrecen, Hungary; daniel.sarkozy@med.unideb.hu; 2 Translational Glycomics Group, Research Institute of Biomolecular and Chemical Engineering, University of Pannonia, 10 Egyetem Street, H-8200 Veszprem, Hungary

**Keywords:** capillary electrophoresis, agarose, SDS-protein complexes, therapeutic antibody

## Abstract

Capillary sodium dodecyl sulfate gel electrophoresis has long been used for the analysis of proteins, mostly either with entangled polymer networks or translationally cross-linked gels. In this paper capillary agarose gel electrophoresis is introduced for the separation of low molecular weight immunoglobulin subunits. The light (LC~24 kDa) and heavy (HC~50 kDa) chain fragments of a monoclonal antibody therapeutic drug were used to optimize the sieving matrix composition of the agarose/Tris-borate-EDTA (TBE) systems. The agarose and boric acid contents were systematically varied between 0.2–1.0% and 320–640 mM, respectively. The influence of several physical parameters such as viscosity and electroosmotic flow were also investigated, the latter to shed light on its effect on the electrokinetic injection bias. Three dimensional Ferguson plots were utilized to better understand the sieving performance of the various agarose/TBE ratio gels, especially relying on their slope (retardation coefficient, K_R_) value differences. The best resolution between the LC and non-glycosylated HC IgG subunits was obtained by utilizing the molecular sieving effect of the 1% agarose/320 mM boric acid composition (ΔK_R_ = 0.035). On the other hand, the 0.8% agarose/640 mM boric acid gel showed the highest separation power between the similar molecular weight, but different surface charge density non-glycosylated HC and HC fragments (ΔK_R_ = 0.005). It is important to note that the agarose-based gel-buffer systems did not require any capillary regeneration steps between runs other than simple replenishment of the sieving matrix, significantly speeding up analysis cycle time.

## 1. Introduction

Agarose is a linear polysaccharide consisting of repeating agarobiose units and a routinely used electrophoresis separation medium in bioanalytical and molecular biology laboratories. With advantageous physicochemical properties, agarose is one of the two major components of a mixture called agar and extracted from red algae by boiling, filtration, and freeze-thawing to remove impurities and the other main component of agaropectin [1,2]. As early as in 1949, Gordon et al. used agar jelly for protein electrophoresis to separate ferritin from hemoglobin and to resolve egg white proteins [3]. However, protein electrophoresis in unprocessed agar was most often compromised by adsorption of sample particles to, or precipitation in the gel [4]. These undesirable properties diminished when the agaropectin and other impurities were removed from the agar. The gels made from the residual agarose were more robust and had significantly less electroosmotic properties [5]. Agarose itself, as the least charged agar subcomponent, was first used to form gels for electrophoresis by Hjerten, who demonstrated strong adsorption of crystal violet in highly purified rein-agar via column electrophoresis, while the extracted agarose showed no detectable interaction with the dye molecule [6]. Since then, agarose has been an extensively used sieving matrix for size separation of biopolymers, mostly DNA but also for proteins. In this latter case, both for analytical and preparative SDS-gel separation of large proteins and protein complexes with the consideration that agarose does not offer adequate separation selectivity for small (<50 kDa) proteins [7]. However, the use of agarose gels in narrow bore capillary columns alleviated this drawback, as shown in this publication.

**Scheme 1:**
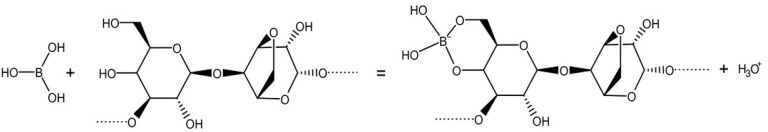
Suggested complexation scheme between the galactose–anhydrogalactose copolymer (agarose) and boric acid.

The primary structure of purified agarose is comprised by alternating galactose-anhydrogalactose building blocks (Scheme 1), sometimes with sulfate, pyruvate and methoxy group substitutions. At neutral pH, these negatively charged residues form a diffuse double layer along the polymer strands, and upon the application of an electric field electroosmotic flow is developed. In case of filling the agarose matrix into narrow bore columns (<100 μm i.d.), this flow is superimposed to the bulk electroosmotic flow of the capillary wall towards the negative electrode (cathode). In his early groundbreaking paper, Serwer suggested that single agarose strands can be arranged in a double helical secondary structure during gelation [8]. However, only shorter agarose chains are capable to produce a sieving matrix with concentration dependent pores sizes. Later, Righetti successfully applied the Ferguson plot method to estimate the apparent pore radius of agarose gels of various concentrations [9].

Capillary SDS gel electrophoresis (SDS-CGE) is one of the mostly used routine purity and release testing methods in the biopharmaceutical industry for protein therapeutics [10] and considered as an automated alternative to SDS polyacrylamide slab gel electrophoresis (SDS-PAGE) [11]. SDS–protein complexes are “free draining” macromolecules with independent electrophoretic mobilities of their molecular weight, due to their practically identical surface charge density, similar to DNA fragments [12,13]. This does not apply for proteins with size and/or charge influencing post translational modifications, such as glycosylation, in which case the glycan moiety does not bind SDS (resulting in less charge) but makes the molecule bulkier (resulting in greater size). A good example of such molecules is the glycosylated heavy chain fragment (HC) of immunoglobulin G (IgG) [14], in which case the Ferguson method may provide adequate molecular mass estimate [15]. In the past, mostly entangled polymer solutions [16] and transitionally cross-linked dextran matrices [14,15,16,17] were used for the separation of SDS–protein complexes in narrow bore capillaries. On this latter, the Mitchelson group published a comprehensive review on the advantages and complexation properties of tetraborate-based gel buffer systems with polyhydroxy (polyol) molecules [18], applicable to agarose too [19].

In this paper we evaluate the effect of agarose/borate concentration ratios on the electromigration properties of SDS–protein complexes, using the low molecular weight light and heavy chain subunits of a monoclonal antibody drug as model compounds. Three dimensional Ferguson plots were generated to understand the migration behavior of the solute molecules for more than a dozen gel formulations and optimize the gel-buffer system.

## 2. Results and Discussion

Capillary SDS agarose gel electrophoresis is introduced for rapid and high-resolution analysis of the low molecular weight subunits of omalizumab (a therapeutic monoclonal antibody sample) with Tris–borate–EDTA (TBE)-based background electrolytes. The agarose and borate concentrations were both varied for separation optimization. The EOF corrected effective electrophoretic mobility values were used to generate three dimensional Ferguson plots to better visualize the sieving behavior of the borate stabilized agarose gels.

### 2.1. The Background Electrolyte

Choosing the appropriate background electrolyte for capillary gel electrophoresis is key in order to achieve rapid and high-resolution separations. Agarose slab gel electrophoresis traditionally employs Tris-based buffer systems mostly with borate, acetate or glycine co-ions, depending on the application in hand. Considering the results of our previous study on dextran-based SDS-CGE separation matrices in narrow bore capillaries [14], first it was important to understand whether the boric acid/agarose (galactose–anhydrogalactose copolymer [20]) complex suggested in Scheme 1 can improve the separation efficiency of SDS–proteins.

**Figure 1:**
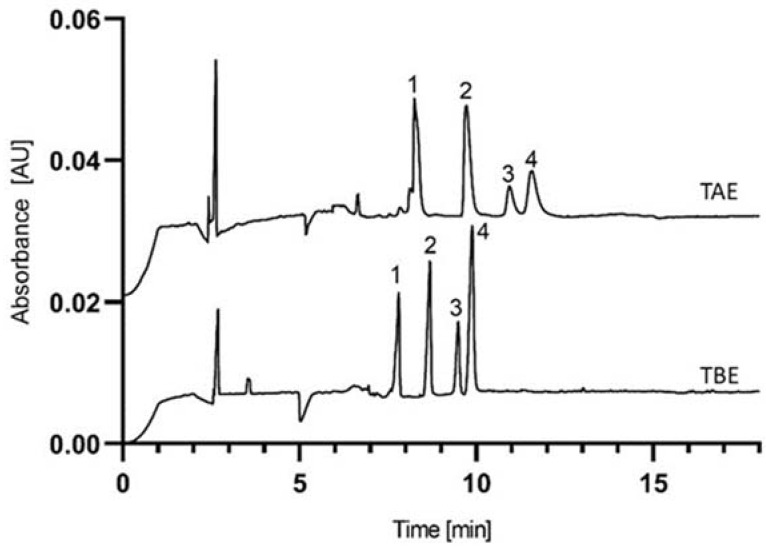
Capillary SDS agarose gel electrophoresis separation of the omalizumab subunits in Tris–acetate–EDTA (TAE, upper trace) and Tris–borate–EDTA (TBE, lower trace) background electrolytes. Peaks: 1–10 kDa internal standard; 2–LC; 3–ngHC; 4–HC. Conditions: bare fused silica capillary (20 cm effective length, 30 cm total length, 50 μm i.d.); UV detection at 214 nm; E = 500 V/cm in reversed polarity (cathode at the injection side); Separation temperature: 25 °C; Electrokinetic injection: 5 kV for 10 s.

In this part of the study, we compared the separation of the SDS complexed omalizumab subunits of light chain (LC), non-glycosylated heavy chain (ngHC) and heavy chain (HC) fragments with the use of Tris–acetate–EDTA (TAE) and Tris–borate–EDTA (TBE)-based buffer systems in 1% agarose. The Tris counter-ion concentration of the background electrolytes were identical in both instances (428 mM) and the pH values were adjusted by boric acid or acetic acid (co-ions), respectively. As Figure 1 shows, the TAE buffer-based separation (upper trace) took somewhat longer with significantly lower peak efficiency (N) and poorer resolution (Rs) than that of with the TBE-based gel-buffer system, in spite of the higher apparent selectivity (α) values with the former (Table 1). This incongruity can be explained by the resolution equation Equation (1), derived earlier for capillary gel electrophoresis of enantiomers by Karger and co-workers [21] and recently applied to SDS-CGE [22].

(1) $$Rs=\frac{1}{\;4}\;\left(\frac{\alpha -1}{\;\alpha }\right)\sqrt{N}\;K$$

where *K* is electrophoretic mobility (μ), applied electric field strength (E) and effective capillary length (ℓ) dependent. Based on Equation (1), resolution is practically assumed to be the outcome of the interplay between selectivity and peak efficiency. In this instance the significantly higher N values obtained with the TBE buffer resulted in better resolutions over the higher selectivity but low efficiency TAE buffer.

**Figure 2:**
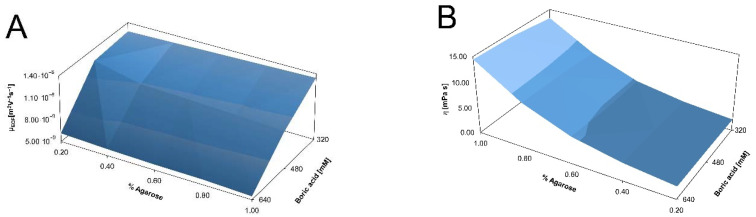
Three-dimensional electroosmotic flow (μ_EOF_, panel **A**) and viscosity (η, panel **B**) plots of the different agarose-TBE gel compositions.

The longer migration time in the TAE-based system was due to the almost twice as high counter-current electroosmotic flow (EOF_TAE_ = 9.15 × 10^−9^ m^2^/Vs vs. EOF_TBE_ = 5.45 × 10^−9^ m^2^/Vs). However, the moderate migration time difference between the two traces (~90 sec) was probably owed to the higher viscosity of the borate stabilized agarose gel (η_TAE_ = 10.04 mPa·s vs. η_TBE_ = 14.45 mPa·s), slowing down electromigration. To better understand this phenomenon, Figure 2 shows the three-dimensional electroosmotic flow (panel A) and viscosity (panel B) plots of the various agarose-TBE gel compositions used in this study. As one can observe, with the agarose-based sieving matrices, the counter current EOF (i.e., moving in the opposite direction to the negatively charged sample components) was mainly dependent on the boric acid concentration (ionic strength) and only marginally on the agarose concentration. The 3D viscosity plot showed the opposite—i.e., reliant mainly on the agarose concentration, but very little on the borate concentration.

Another interesting observation was that in the case of using TAE as background electrolyte, decreasing peak heights were detected with increasing MW (main peaks 1, 2 and 4), while the opposite peak height distribution was obtained with the TBE-based buffer system. We considered this effect as an injection artefact, i.e., the consequence of the ~2× greater countercurrent electroosmotic flow in the TAE buffer that influenced the electrokinetic sample introduction bias. The sharper peaks obtained with the TBE-based buffer system (Table 1, column N), on the other hand, was considered to be the result of the borate complexation with the galactose constituents of the agarose as shown in Scheme 1, and the concomitant pore size adjustment mediated better sieving capability, considering galactose-borate di-diol complex-based cross-linking [23]. Therefore, TBE-based background electrolytes were chosen for all downstream experiments with the agarose gels.

### 2.2. Separation of the SDS-Protein Complexes

**Figure 3:**
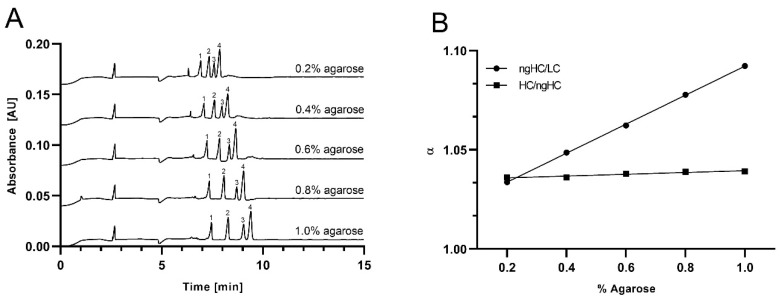
Separation of the SDS-omalizumab subunit complexes in increasing agarose concentration gels with 640 mM TBE background electrolyte (**A**). The agarose concentration-based selectivity changes between the ngHC/LC, and HC/ngHC fragments are shown in panel (**B**). Peak assignment and separation conditions were the same as in Figure 1.

**Figure 4:**
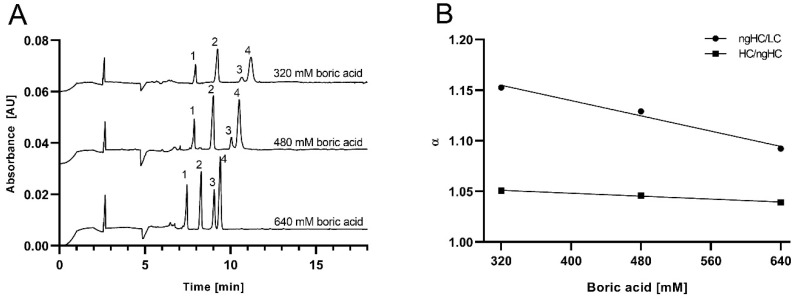
Separation of the SDS-omalizumab subunit complexes in 1% agarose gels with increasing TBE background electrolyte concentrations (**A**). The selectivity changes as the function of boric acid concentration between the ngHC/LC and HC/ngHC fragments are shown in panel (**B**). Peak assignment and separation conditions were the same as in Figure 1.

To understand the migration behavior of the omalizumab subunits in agarose–TBE based gels, the agarose concentration was evaluated in the range of 0.2% to 1.0% in 0.2% increments with 320, 480 and 640 mM boric acid containing background electrolytes, all adjusted to pH 8.0 by Tris base. The separation results of the various agarose–TBE concentration combinations used for the analysis of the low molecular weight monoclonal antibody fragments are summarized in Table 2 and by the representative examples in Figure 3 and Figure 4.

First, the effect of agarose concentration was evaluated on the separations and an example is shown with the 640 mM TBE containing background electrolyte in Figure 3A. As expected, in this instance the mobilities of the sample components decreased with increasing gel concentration, and with concomitantly increasing viscosity (Figure 2, panel B). With elevating agarose concentration levels, the selectivities between the non-glycosylated heavy chain (ngHC) and the heavy chain (HC) fragments practically did not change (Figure 3B, HC/ngHC), however, significantly increased between the light chain (LC) and the non-glycosylated heavy chain (ngHC) fragments (Figure 3B, LC/ngHC and Table 2). This phenomenon was probably the result of the glycosylation mediated surface charge density differences of the similar size ngHC and HC (cc 48 vs. 50 kDa) fragments [15] in contrast to the same surface charge density but different size LC and ngHC (cc 24 vs. 48 kDa) subunits. In other words, in the instance of the ngHC/HC peak pair, the separation was probably based on their hydrodynamic volume to charge ratio, i.e., apparently independent of the agarose concentration, in contrast to the latter case where the sieving effect of the agarose gel played a key role.

Figure 4A shows the separation traces using 1% agarose with the three different TBE buffer concentrations specified above. Due to the decreasing EOF with increasing background electrolyte concentration (Figure 2, panel A), the apparent electrophoretic mobilities of the sample components increased. The resulting separation characteristics, such as selectivity, theoretical plate number and resolution are listed in Table 2. Figure 4B delineates the co-ion concentration-based selectivity dependence for the LC/ngHC and ngHC/HC peak pairs, both decreasing at different rates with increasing borate concentration. This outcome was probably due to the increasing counter current EOF at lower borate concentrations, having the same effect as if one would use longer and longer capillary columns for the separations. Practically with all agarose concentrations, the highest theoretical plate numbers were achieved with the 640 mM TBE buffer, while the best selectivities were observed with the 320 mM borate containing background electrolyte (Table 2). Here, in spite of the lower theoretical plate numbers offered by the 320 mM borate buffer containing gels, the best resolution values were obtained for the LC/ngHC pair, due to the highest separation selectivities Equation (1). Interestingly, for the ngHC/HC pair, best resolution was attained with the interim gel concentration of 0.8% agarose with 640 mM borate. As was emphasized above, for this peak pair, the separation was mainly dependent on the surface charge density differences between these very close MW fragments as shown later numerically in Section 2.3. The relative peak heights of the higher MW subunits increased with increasing TBE concentration due to the concomitantly decreasing EOF. This phenomenon must have been caused by a similar injection bias alteration as discussed above in Section 2.1.

The total separation time difference between the 320 mM (upper trace) and 640 mM (lower trace) borate containing background electrolytes was only <1.5 min. Importantly, no in-between-runs capillary regeneration steps were necessary with the agarose-TBE gel-buffer composition, still attaining excellent (<0.75% RSD) migration time reproducibility over the course of 10 consecutive runs.

### 2.3. Ferguson Plots of Capillary SDS Agarose Gel Electrophoresis of Proteins

Electric field mediated differential migration of SDS-proteins in gels assumes spherical-shape species moving across the porous sieving matrix. Therefore, a linear relationship can be considered between the logarithmic effective electrophoretic mobility (μ_eff_) and the gel concentration (T) (Ferguson plot [24]) as defined by Equation (2):

log μ_eff_ = log μ_eff_^0^_−_K_R_T (2)

(μ_eff_^0^ represents the free solution mobility and K_R_ the retardation coefficient). To calculate the effective electrophoretic mobilities (μ_eff_) for the Ferguson plots, the countercurrent electroosmotic flow mobilities (μ_EOF_) were algebraically summed with the apparent electrophoretic mobilities (μ_app_) of the sample components:

μ_eff_ = μ_app_ + μ_EOF_ (3)

**Figure 5:**
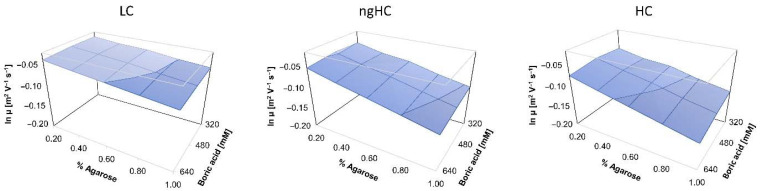
Three-dimensional Ferguson plots for the light (panel LC), non-glycosylated heavy (panel ngHC) and heavy (panel HC) chain subunits of omalizumab.

The 3D logarithmic mobility vs. gel concentration surfaces were plotted for the light chain (panel LC), non-glycosylated heavy chain (panel ngHC) and heavy chain (panel HC) subunits of omalizumab in Figure 5. As shown in the three panels, with increasing borate concentration the mobilities of the subunits did not significantly change at the 0.2% agarose concentration level but revealed slightly decreasing tendency with the 1.0% agarose containing sieving matrix. On the other hand, with increasing agarose concentration, linearly decreasing plots were obtained for all three SDS–protein fragments at all boric acid concentrations, unlike reported earlier for dextran-based gels, in which case concave surfaces were observed [25]. The average slope values, generally referred to as retardation coefficient (K_R_, Equation (2)), for the LC, ngHC and HC fragments were −0.03, −0.065 and −0.07, respectively.

Based on these Ferguson plot slope values and the data shown in Table 2, best separation performance between the LC and ngHC subunits can be attained with the use of 1% agarose/320 mM boric acid containing background electrolyte yielding the highest resolution with short (<11 min) analysis times. The resolution between the ngHC and HC fragments was apparently not size but surface charge density dependent as suggested by their almost parallel K_R_ values of −0.065 vs. −0.07, with the best resolution obtained by applying the 0.8% agarose/640 mM borate containing gel composition.

## 3. Conclusions

In this paper, capillary SDS agarose gel electrophoresis was introduced for the analysis SDS–protein complexes using Tris−borate−EDTA containing background electrolytes, exploiting the tetraborate adduct-based stabilization via the galactose constituents of the agarose chains. The low molecular weight subunit sample components of a monoclonal antibody drug (omalizumab) were utilized for sieving matrix composition optimization. First the performance of acetate (TAE) and borate (TBE)-based buffer systems were compared, the latter showing better separation characteristics. Different agarose (0.2–1.0%) and the boric acid (320–640 mM) concentrations were evaluated to optimize the gel-buffer system. The viscosity and electroosmotic flow of the sieving gel compositions were also defined, this latter to better understand the EOF mediated alterations of the electrokinetic injection bias and the concomitant peak size distribution alterations. Three-dimensional Ferguson plots were graphed, including all fifteen agarose-TBE gel compositions evaluated in this study, and their slopes provided the retardation coefficients (K_R_, Equation (2)). The best separation performance between the LC (non-glycosylated) and ngHC fragments was obtained with the 1% agarose/320 mM boric acid gel-buffer system exhibiting mostly molecular sieving by the Ferguson plots (K_R_ = −0.03 vs. −0.065, Δ_KR_ = 0.035). The glycosylated HC, on the other hand, was best resolved from its ngHC counterpart by the 640 mM borate containing 0.8% agarose gel, probably based on surface charge density differences rather than size (K_R_ = −0.065 vs. −0.07, Δ_KR_ = 0.005). In addition to the short separation times of <11 min, another advantage of agarose-based gels is to be able to skip the otherwise important capillary regeneration steps, required by other SDS sieving matrices between each run. Simple replenishment of the borate stabilized agarose gel remarkably speeded up analysis turnaround times with <0.75% migration time RSD.

## 4. Materials and Methods

### 4.1. Chemicals and Reagents

Ultra-low gelling temperature agarose, sodium dodecyl sulfate (SDS), boric acid, Tris, EDTA·Na_2_, mesityl oxide, sodium hydroxide, hydrochloric acid, HPLC grade water, glacial acetic acid, glycerol and 2-mercaptoethanol were purchased from Sigma Aldrich (St. Louis, MO, USA). The 10 kDa size standard and the SDS-MW sample buffer were from Bio-Science Kft (Budapest, Hungary). The PNGase F enzyme was from the Bio-Nanosystems Laboratory, University of Pannonia (Hungary). The therapeutic monoclonal antibody drug (omalizumab, 10 mg/mL) was a kind gift of the Borsod Academic County Hospital (Miskolc, Hungary).

### 4.2. Agarose Gel and Sample Preparation

The background electrolytes for the agarose gel–Tris–borate–EDTA (TBE) buffer compositions consisted of increasing amounts of boric acid (320, 480 and 640 mM) adjusted to pH 8.0 with Tris base, followed by the addition of 2 mM of EDTA·Na_2_ and 10% (*v*/*v*) of glycerol. Then, the ultra-low gelling temperature agarose was added in the amounts to obtain 0.2, 0.4, 0.6, 0.8 and 1.0% (*w*/*v*) final concentrations, resulting in this way 5 different gel compositions for the three boric acid concentration background electrolytes (15 compositions total). After overnight stirring at 75 °C (250 RPM using a magnetic hotplate). 0.2% (*w*/*v*) SDS was added and mixed at room temperature slowly (at ~100 RPM) for an additional hour to prevent foaming. For the Tris–acetate–EDTA (TAE) buffer, 428 mM Tris base solution was used (the same as for TBE) and the pH was adjusted to 8.0 with glacial acetic acid. Every other component concentration and preparation steps were the same as for the borate containing gel-buffers.

The sample was prepared by adding 100 mg omalizumab (10 μL of 10 mg/mL stock solution) to 80 μL of sample buffer, 2 μL of 10 kDa internal standard solution and 5 μL of 2–mercaptoethanol, mixed in a PCR tube. For *N*–glycan removal from the heavy chain of the IgG, the sample was first denatured by using a gradient temperature protocol [26] to minimize fragmentation artifacts, then PNGase F (200 mU) digestion was applied at 50 °C for 1 h.

### 4.3. Capillary Agarose Gel Electrophoresis

In all capillary agarose gel electrophoresis separations, a P/ACE MDQ Capillary Electrophoresis System (Beckman Coulter, Brea, CA) was used with UV absorbance detection (214 nm). The separations were accomplished in 20 cm effective length (30 cm total length), 50 μm i.d. bare fused silica capillaries, conditioned by rinsing with 0.1 M NaOH for 3 min, 0.1 M HCl for 1 min and HPLC grade water for 5 min before the first use of the column. The capillary was then filled with the respective gel-buffer system (60 psi rinse pressure for 5 min) before each run. With the use of higher agarose concentration compositions (0.8 and 1.0% *w*/*v*) a capillary conditioning step is recommended after each 10 runs. Reversed polarity (cathode at the injection side) separation mode was used by applying −15 kV electric potential at 25 °C. The samples were electrokinetically injected for 10 s at 5 kV. The PeakFit (version 4.12) software (sytat.com) was used for data processing and analysis.

### 4.4. Electroosmotic Flow and Viscosity Measurements

All electroosmotic flow (EOF) and viscosity measurements were made in triplicates with UV detection at 254 nm wavelength. The separation capillary was conditioned as described above and loaded with the respective gel buffers. The mesityl oxide EOF marker (50 mM in HPLC-grade water) was injected for 20 s at 5 kV. Then +15 kV electric potential (normal polarity) was applied at 25 °C as at the neutral pH of the background electrolytes the EOF was cathodic. The electroosmotic flow values in all gel-buffer compositions were calculated based on the migration time of mesityl oxide. The viscosities of the gel-buffer systems were measured by pressure injecting the mesityl oxide (5 psi/5 s) and applying 20 psi forward pressure to mobilize the sample towards the detection window. Viscosity values were calculated using the Hagen-Poiseuille equation as described in [27].

## Figures and Tables

**Table 1:** Comparison of the separation characteristics (α–selectivity, *N*-theoretical plate number, Rs–resolution) with agarose–TAE and agarose-TBE gel-buffer systems.

Peak	α		N			Rs	
	ngHC/LC	HC/ngHC	LC	ngHC	HC	ngHC/LC	HC/ngHC
**Agarose-TAE**	1.140	1.055	15967	10579	6455	3.000	0.962
**Agarose-TBE**	1.092	1.039	39316	45012	32201	3.435	1.505

**Table 2:** Separation selectivity (α), efficiency (N) and resolution (Rs) values with the agarose-TBE gel-buffers evaluated in this study.

%Agarose	320 mM Boric acid						
	α		N			Rs	
	ngHC/LC	HC/ngHC	LC	ngHC	HC	ngHC/LC	HC/ngHC
**0.2**	1.048	1.049	22321	31125	16204	1.504	1.450
**0.4**	1.073	1.051	27609	29863	22158	2.476	1.593
**0.6**	1.088	1.045	29155	30386	19964	3.799	1.545
**0.8**	1.124	1.051	29528	24799	20088	4.051	1.580
**1.0**	1.153	1.051	22114	18238	12858	4.319	1.254
**%Agarose**	**480 mM Boric acid**						
	**α**		**N**			**Rs**	
	**ngHC/LC**	**HC/ngHC**	**LC**	**ngHC**	**HC**	**ngHC/LC**	**HC/ngHC**
**0.2**	1.043	1.050	22755	38553	21541	1.374	1.440
**0.4**	1.063	1.045	27691	31503	22210	1.718	1.318
**0.6**	1.082	1.044	29063	30346	25263	2.789	1.581
**0.8**	1.108	1.048	30118	34139	28627	3.931	1.602
**1.0**	1.129	1.046	27624	39007	19911	3.939	1.536
**%Agarose**	**640 mM Boric acid**						
	**α**		**N**			**Rs**	
	**ngHC/LC**	**HC/ngHC**	**LC**	**ngHC**	**HC**	**ngHC/LC**	**HC/ngHC**
**0.2**	1.034	1.036	32775	52624	29885	1.170	1.342
**0.4**	1.049	1.036	33757	50221	28982	1.811	1.292
**0.6**	1.062	1.038	34210	48996	29605	2.155	1.389
**0.8**	1.078	1.039	32818	66250	34239	3.032	1.653
**1.0**	1.092	1.039	39316	45012	32201	3.435	1.505

## Data Availability

The data presented in this study are available on request from the corresponding author.

## Abbreviations

3D	three dimensional
CGE	capillary gel electrophoresis
EOF	electroosmotic flow
HC	heavy chain
i.d.	internal diameter
LC	light chain
N	theoretical plate number
ngHC	non-glycosylated heavy chain
Rs	resolution
SDS	sodium dodecyl sulfate
TAE	Tris
acetate	EDTA
TBE	Tris
borate	EDTA
α	selectivity
